# A Case Control Study of the Seroprevalence of *Helicobacter pylori* Proteins and Their Association with Pancreatic Cancer Risk

**DOI:** 10.1089/pancan.2021.0010

**Published:** 2021-09-16

**Authors:** Jennifer B. Permuth, Shams Rahman, Dung-Tsa Chen, Tim Waterboer, Anna R. Giuliano

**Affiliations:** ^1^ Department of Cancer Epidemiology, H. Lee Moffitt Cancer Center and Research Institute, Tampa, Florida, USA.; ^2^ Department of Public Health and Health Equity, College of Nursing and Health Sciences, Bethune-Cookman University, Daytona, Florida, USA.; ^3^ Department of Biostatistics and Bioinformatics, H. Lee Moffitt Cancer Center and Research Institute, Tampa, Florida, USA.; ^4^ Division of Infections and Cancer Epidemiology, German Cancer Research Center, Heidelberg, Germany.; ^5^ Center of Infection Research in Cancer, H. Lee Moffitt Cancer Center and Research Institute, Tampa, Florida, USA.

**Keywords:** biomarkers, cancer, *H. pylori*, pancreas, seroepidemiology

## Abstract

**Background:** The association between *Helicobacter pylori* (*H. pylori*) infection and pancreatic cancer (PC) risk remains inconclusive. We examined the association between *H. pylori* antibodies and PC risk in a case-control study at a comprehensive cancer center.

**Methods:** Multiplex serology using a glutathione S-transferase capture immunosorbent assay in conjunction with fluorescent bead technology was used to measure antibodies to 15 *H. pylori* proteins in serum or plasma from 131 incident cases with PC or a PC precursor and 131 healthy controls. Reactivity to ≥4 *H. pylori* proteins was defined as the overall seroprevalence. Logistic regression was used to calculate odds ratios (ORs) and 95% confidence intervals (CIs), with adjustment for age at diagnosis/interview, gender, and race.

**Results:** The majority of the sample was 50 years or older, and from the white race group. Half of the sample were women. Seroprevalence ≥4 of *H. pylori* proteins was 11.1%. Overall, *H. pylori* seroprevalence was not associated with PC risk (OR: 0.59; 95% CI: 0.25–1.40). The prevalence of several *H. pylori*-specific proteins HP537 (OR: 1.78; 95% CI: 0.30–10.51), HP305 (OR: 1.38; 95% CI: 0.61–3.16), and HP410 (OR: 1.31; 95% CI: 0.44–3.96) increased the odds of PC. Similarly, *H. pylori*-specific proteins HP522 (OR: 0.25; 95% CI: 0.04–1.66), HyuA (OR: 0.49; 95% CI: 0.21–1.14), and HP1564 (OR: 0.63; 95% CI: 0.27–1.51) decreased the odds of PC. However, these findings were not statistically significant at *α* = 0.05.

**Conclusions:** Our findings do not support an association between *H. pylori* and PC risk. Further evaluation of this lack of association is recommended.

## Introduction

Pancreatic cancer (PC) is the third leading cause of cancer mortality in the United States.^1^ The vast majority of PC cases are diagnosed at an advanced stage, in which ∼8–9% of people survive only 5 years.^2^ The etiology of PC is not sufficiently understood.

Up to 40% of PC cases are attributable to established risk factors such as tobacco exposure, increased age, familial history of the disease, diabetes mellitus, chronic pancreatitis, obesity, and ABO blood group.^3^ Based on an emerging body of evidence in support of infectious agents as contributors to PC risk,^3–8^ there is a rationale for studying the role of infectious agents in the etiology of PC.

*Helicobacter pylori* infection promotes chronic inflammation in the gastric mucosa and is the strongest risk factor for gastric cancer.^9^ It has been hypothesized that this bacterium may also influence pancreatic carcinogenesis through pathophysiological actions summarized by Risch.^5^ In brief, colonization of the gastric antrum may reduce the number of antral D-cells and suppress the production of somatostatin. This promotes hyperacidity (depending on the strain), resulting in an increase in secretion of secretin and pancreatic bicarbonate output. Secretin has been shown to promote murine pancreatic growth and DNA synthesis in PC cells, and it is possible that induced ductal epithelial cell proliferation could enhance the effect of known carcinogens such as N-nitrosoamines in the pancreas, leading to PC.^10,11^ Interactions with ABO genotype, dietary, and smoking habits may impact these processes.^5^ Although some studies^12,13^ have not been able to isolate *H. pylori* DNA from pancreatic tumor tissues or pancreatic juice, others have.^14^ Nilsson et al. evaluated several *Helicobacter* species and found that 75% (30/40) of exocrine PC tumors were *Helicobacter* positive (with *H. pylori*, *flexispira*, or *cineadi*) compared to 60% of chronic pancreatitis cases and 0% of controls (normal pancreas tissue and pancreatic cysts).^14^ Finally, *in vitro* investigations suggest that *H. pylori* infection of human PC cells may increase their malignant potential by inducing increases in IL-8 and VEGF secretion levels and activities of proliferation factors NF-κB, AP1, and SRE.^15^

Several meta-analyses have evaluated the association between *H. pylori* infection and PC risk with mixed results. Some studies did not find an association with odds ratio (OR): 0.99; 95% confidence interval (CI): 0.65–1.50 and OR: 1.06; 95% CI: 0.74–1.37 and OR: 1.13; 95% CI: 0.86–1.50.^8,16,17^ Other studies reported increased risk for *H. pylori*-positive subjects with OR 1.38, 95% CI: 1.08–1.75; OR: 1.45 (95% CI: 1.09–1.92 and OR: 1.47; 95% CI: 1.22–1.77.^18–20^ The meta-analysis that included the most studies included 10 studies (6 nested case-control, 3 case-control; and 1 cohort) that used IgG enzyme-linked immunosorbent assay (ELISA) kits for serological analysis.^8^ Although there was no significant overall association between *H. pylori* seropositivity and PC risk (OR: 1.13; 95% CI: 0.86–1.50), specific associations were observed for antibodies against the cytotoxin-associated gene A (CagA) protein encoded on the cag pathogenicity island, which distinguishes the more strongly cancer-associated type I strains (CagA^+^) from the less carcinogenic type II (CagA^−^) strains (CagA^+^: OR 0.78; 95% CI: 0.67–0.91 vs. CagA^−^ OR: 1.30 95% CI: 1.02–1.65). The association with CagA^−^ strains was largely driven by the Risch et al. study,^7^ who were the first to study strain-specific associations between *H. pylori* and PC risk in the United States among 373 PC cases and 690 controls from Connecticut. They showed that seropositivity for CagA^−^ *H. pylori* strains could be a risk factor for PC, especially among those with a non-O blood group (OR: 2.78; 95% CI: 1.49–5.20).^7^ It has been suggested that there may be possible differences in terminal binding antigens in gastrointestinal mucins for individuals with non-O blood groups (A and B).^5,7^

A population-based case-control study in China reported an increased, but nonsignificant, risk of PC for CagA^−^ *H. pylori* seropositivity (OR: 1.28; 95% CI: 0.76–2.13).^6^ Another small pilot study in China^21^ showed that individuals who were serum CagA^+^ via ELISA have a higher risk of PC; blood group was not evaluated in these studies.^6,21^ More recently, Liu et al.^22^ conducted a meta-analysis of 65,155 observations from nine prospective epidemiologic studies (three prospective cohorts and six nested case-control studies), an association between *H. pylori* infection and PC risk was not identified (OR: 1.09; 95% CI: 0.87–1.47). An analysis of CagA strains of *H. pylori* showed a positive association (OR: 1.30; 95% CI: 1.05–1.62),^22^ but the association did not persist after a sensitivity analysis, which excluded the study by Risch et al.^7^ Contrary to a prior ELISA-based study, in which Stolzenberg-Solomon and colleagues^23^ detected an association between *H. pylori* and PC risk (OR: 1.87; 95% CI: 1.05–3.34) in a cohort of Finnish male smokers from the Alpha-Tocopherol, Beta-Carotene Cancer Prevention Study (ATBC), Yu et al.^24^ later found no association between *H. pylori* seropositivity and PC risk using a multiplex serology assay in a larger case-control study of 353 PC cases and 353 controls nested within the ATBC cohort who were matched on date of baseline serum collection, age at randomization, and follow-up time (overall seropositivity OR: 0.85; 95% CI: 0.49–1.49). Discrepancies may be explained by differences in the technology used.

Possible publication bias has been detected in some meta-analyses,^19^ and it has been suggested that additional case-control and cohort studies are needed to confirm or refute observed associations with PC risk. Furthermore, additional strains of *H. pylori* warrant investigation since most of the prior studies in this area did not take into account a wide array of *H. pylori* strains. As such, the objective of this investigation was to conduct the first case-control study of PC in the United States to determine whether an association exists between *H. pylori* seropositivity and PC risk in males and females using 15 different multiplex serology antigens: GroEL (HP10), UreA (HP73), HP0231, NapA (HP243), HP0305, HpA (HP410), Cag delta (HP522), CagM (HP537), CagA, HyuA, Catalase (HP875), VacA, HcpC (HP1098), Cad (HP1104), and HP1564.

## Materials and Methods

### Study population and biospecimens

A prospectively maintained clinical database was retrospectively reviewed to identify individuals who were diagnosed and treated for PC (or premalignant pancreatic cystic lesions) between 2004 and 2015 at Moffitt Cancer Center and Research Institute located in Tampa, FL. Patients had provided written consent for blood to be donated preoperatively for research through Institutional Review Board (IRB) protocols, including Moffitt's Total Cancer Care (TCC) Protocol (MCC 14690/IRB 104189), pre-HIPAA protocols, and a Moffitt General Banking Protocol entitled “Procurement, Banking, and Release of Residual Human Biological Materials for Research” (MCC 13579/IRB 101642). IRB approval was specifically granted for the research described herein (IRB#Pro4971). The diagnosis of cases was histologically confirmed. None of the cases received preoperative chemotherapy or radiation. Simultaneously, through Moffitt's Lifetime Database for Cancer Risk Assessment and Early Detection Protocol (MCC 14453/IRB 103792), gender-matched healthy controls with no current or prior history of pancreatic disease or symptoms who donated blood samples for future research purposes were also recruited.

Blood was collected from consented participants via phlebotomy in 7-mL tubes; ethylenediaminetetraacetic acid (EDTA) tubes were processed for plasma and serum, separator tubes were processed for serum within 2 h using standard procedures. More specifically, EDTA tubes were inverted three times and spun at 3,600 rpm for 8 min and red top tubes sat for 30 min to allow clotting and then underwent centrifugation @1300 *g*/RT/10 min. Samples were aliquoted into 0.5 mL cryovials and stored at −80°C. Correlative demographic, clinical, and epidemiologic data were collected from an electronic questionnaire, the medical record, Moffitt's cancer registry, and other source systems.

### *H. pylori* multiplex serology

For each case, ∼50 μL of plasma or serum were retrieved and shipped to the German Cancer Research Center for analysis. The laboratory simultaneously measured antibodies against the following 15 *H. pylori* proteins: Cad (cinnamyl-alcohol-dehydrogenase ELI3–2), Cag delta (cag pathogenicity island protein δ), CagM (cag pathogenicity island protein M), CagA (cytotoxin-associated gene A), Catalase, HcpC (conserved hypothetical secreted protein-paralog HcpA induces IFNγ), HP0231 (hypothetical protein HP0231), HP0305 (hypothetical protein HP0305), HpaA (neuraminyllactose-binding hemagglutinin homolog), HyuA (hydantoin utilization protein A), GroEL (chaperonin GroEL), NapA (neutrophil activating protein [bacterioferritin]), HP1564, VacA (vacuolating cytotoxin), and UreA (urease alpha subunit). The multiplex assay has been described recently.^25^ In brief, the *H. pylori* multiplex serology uses a glutathione-S-transferase (GST) capture immunosorbent assay in conjunction with fluorescent bead technology (Luminex) to detect a IgA, IgM, and IgG antibodies to 15 *H pylori* proteins.^26,27^ HepatitisB,^28^ p53,^29^ and BK polyomavirus antigens^30^ were also evaluated as specificity controls. The cutoff for seropositivity to each protein was based on median fluorescence intensity for specific proteins in known *H. pylori*-negative sera.

### Statistical analysis

We assessed individual associations of seropositivity to each of the 15 proteins with PC risk using unconditional logistic regression, with adjustment for potential confounders (age, gender, and race). Overall *H. pylori* seroprevalence was defined as reactivity with ≥4 proteins out of the 15 tested for. The ≥4 threshold definition was selected based on a previously reported study that examined the sensitivity and specificity of *H. pylori*-specific GST-based multiplex serology assay.^26^ We also estimated seroprevalence based on CagA *H. pylori* protein.

Due to insufficient volume of specimens available for analysis, four cases and four controls were removed from all analyses. The final sample included 131 cases and 131 controls. Cases and controls were compared for demographic characteristics using chi-square and Fisher exact tests. Logistic regression models were used to examine the associations between seropositivity to 15 *H. pylori* proteins and case-control status. ORs were adjusted for age, gender, and race.

## Results

Demographic characteristics of 131 cases and 131 healthy controls, and histopathologic characteristics for cases are shown in Table 1. Compared with controls, cases were significantly older (mean age 67.6 years vs. 59.0; *p*-value <0.001). Approximately 50% of both cases and controls were males and 50% females. Most of the sample size was white/Caucasian (115 cases and 105 controls). Approximately 93% of the cases were of the pancreatic ductal adenocarcinoma type, and 80.2% were either regional or metastatic. Nine cases (6.9%) had premalignant cystic precursors known as intraductal papillary mucinous neoplasms (IPMNs) or mucinous cystic neoplasms. More than 60% of tumors were in the pancreatic head (Table 1).

**Table 1.** Demographic Characteristics of the Study Participants and Tumor Histopathology of Pancreatic Cancer Cases

	Cases (*N* = 131)		Controls (*N* = 131)			*Helicobacter pylori* positive^a^		*H. pylori* negative^a^		
Characteristic	*n*	%	*N*	%	*p* ^b^	*n*	%	*n*	%	*p* ^b^
Age, years										
≤49	7	23.3	23	76.7	**<0.0001**	2	6.7	28	93.3	0.163
50–59	16	28.6	40	71.4		3	5.4	53	94.6	
≥60	108	61.4	68	38.6		24	13.6	152	86.4	
Gender										
Female	66	50.8	64	49.2	0.8048	14	10.8	116	89.2	0.878
Male	65	49.2	67	50.8		15	11.4	117	88.6	
Race										
White, non-Hispanic	115	52.3	105	47.7	0.0922	18	8.2	202	91.8	**0.002**
Other	16	38.1	26	61.9		11	26.2	31	73.8	
Histology of tumor										
Ductal adenocarcinoma	*122*	*93.1*				12	9.8	110	90.2	0.999
IPMN or MCN	*9*	*6.9*				1	11.1	8	88.9	
Stage of tumor^c^										
Localized (stages: 0, IA, IB)	*24*	*18.3*				2	8.3	22	91.7	0.383
Regional (stages: IIA, IIB, III)	*83*	*63.4*				8	9.6	75	90.4	
Metastatic (stage IV)	*22*	*16.8*				2	9.1	20	90.9	
Missing	*2*	*1.5*				1	50.0	1	50.0	
Differentiation										
Well/moderately differentiated	*45*	*34.4*				6	13.3	39	86.7	0.999
Poorly differentiated/undifferentiated	*19*	*14.5*				3	15.8	16	84.2	
Not determined	*67*	*51.2*				4	6.0	63	94.0	
Tumor location										
Head of pancreas	*82*	*62.6*				5	6.1	77	93.9	0.375
Body of pancreas	*14*	*10.7*				3	21.4	11	78.6	
Tail of pancreas	*23*	*17.6*				4	17.4	19	82.6	
Ducts of pancreas	*1*	*0.8*				0	0.0	1	100	
Pancreas overlapping	*3*	*2.3*				0	0.0	3	100	
Not specified	*8*	*6.0*				1	12.5	7	87.5	

Table shows row percent for cases versus controls, and column percent (italicized) for distribution of a characteristic among cases only.

^a^ Overall HP seropositivity was defined as reactivity with at least 4 proteins out of the 15, listed in the Table 2 that is, column 2 to column 16.

^b^ *p-*Value was estimated using the chi-square or the Fisher exact tests when cells had counts <5. Missing data were excluded from *p*-value calculation. Bold indicates a significant *p*-value.

^c^ Classification is based on the 6th edition of AJCC manual.

IPMN, intraductal papillary mucinous neoplasm; MCN, mucinous cystic neoplasm.

Table 2 shows the seroprevalence of 15 antibodies to *H*. *pylori* proteins and their associations with PC case-control status, with adjustment for age, gender, and race. Approximately 29 (11.1%) of the entire study sample was positive for *H. pylori* ≥4 of the 15 proteins [cases 13 (9.9%) vs. control 16 (12.2%); chi-square *p*-value = 0.555], and 29 (11.1%) were positive for CagA [cases 14 (10.7%) vs. control 15 (11.5%) chi-square *p*-value = 0.844]. Overall, significant associations between seropositivity to each protein and PC risk were not observed. The prevalence of several *H. pylori*-specific proteins (i.e., HP537: OR 1.78; HP305: OR 1.38; and HP410: OR 1.31) increased the odds of PC, but these findings did not reach significance at *α* = 0.05. Similarly, *H. pylori*-specific proteins (i.e., HP522: OR 0.25; HyuA: OR 0.49; and HP1564: OR 0.63) decreased the odds of PC, but these findings did not reach significance at *α* = 0.05. Stratification by or adjustment for family history, diabetes, chronic pancreatitis, obesity, and ABO blood group was not possible because this information was missing for most healthy controls. The seropositivity for p53 was insignificantly higher among cases (OR: 3.12; 95% CI: 0.91–10.75), and the seropositivity for BK VP1 was insignificantly higher among controls (OR: 0.96; 95% CI: 0.51–1.81).

**Table 2.** Seroprevalence for 15 Antibodies to *H. pylori* Proteins and Their Associations with Pancreatic Cancer Case-Control Status

Antibody	Cases (*N* = 131)		Controls (*N* = 131)			
	*n*	% Positive	*n*	% Positive	*p* ^a^	OR (95% CI)^b^
Overall HP seropositivity^c^	13	44.8	16	55.2	0.555	0.59 (0.25–1.40)
HP10	31	49.2	32	50.8	0.885	0.78 (0.41–1.47)
HP73	18	56.3	14	43.7	0.450	1.06 (0.48–2.37)
HP231	8	44.4	10	55.6	0.625	0.67 (0.23–1.90)
HP243	15	51.7	14	48.3	0.849	1.01 (0.43–2.33)
HP305	19	61.3	12	38.7	0.180	1.38 (0.61–3.16)
HP410	9	56.3	7	43.7	0.601	1.31 (0.44–3.96)
HP522	2	28.6	5	71.4	0.447	0.25 (0.04–1.66)
HP537	5	71.4	2	28.6	0.447	1.78 (0.30–10.51)
HP875	14	46.7	16	53.3	0.698	0.69 (0.30–1.59)
HP1098	14	53.8	12	46.2	0.679	0.84 (0.35–2.03)
HP1104	5	62.5	3	37.5	0.722	1.09 (0.23–5.19)
HP1564	14	46.7	16	53.3	0.698	0.63 (0.27–1.51)
CagA	14	48.3	15	51.7	0.844	0.91 (0.37–2.22)
HyuA	11	37.9	18	62.1	0.168	0.49 (0.21–1.14)
VacA	7	53.8	6	46.2	0.776	0.99 (0.29–3.37)
p53	12	75.0	4	25.0	**0.039**	3.12 (0.91–10.75)
BK VP1	101	49.3	104	50.7	0.653	0.96 (0.51–1.81)

^a^ *p*-Value is from chi-square and Fisher exact tests, comparing cases and controls for respective *H. pylori* protein. Bold indicates a significant *p*-value.

^b^ OR; 95% CI. OR compares cases against controls, for each antibody seronegative group was considered as the reference group. ORs were adjusted for age, gender, and race.

^c^ Overall HP seropositivity was defined as reactivity with at least 4 proteins out of the 15, listed in the Table 2 that is, row 2 to 16.

CI, confidence interval; OR, odds ratio.

## Discussion

*H. pylori* is a gram-negative spiral-shaped bacterium known to play a role in contributing to gastric and duodenal ulcers and stomach cancer.^31–34^ The potential role of *H. pylori* has also been suspected in colorectal carcinogenesis although the evidence is not conclusive.^25,35^ PC remains one of the most lethal forms of cancer, and the role of *H. pylori* in the etiology of PC is unclear. Several studies have reported the potential role of *H. pylori* in pancreatic carcinogenesis, with an increased risk among *H. pylori*-CagA-positive individuals, with ORs ranging from 1.38 to 1.65, while others failed to detect associations or only detected an association with CagA-negative strains.^7,8,16–20^ We evaluated associations between the prevalence of 15 *H. pylori*-specific antigens and PC risk.

In line with a robust study of the Finnish ATBC cohort that also evaluated the same antigens and PC risk,^24^ our results were null. In addition, consistent with a recent study, which reported absence of *H. pylori* DNA either in IPMN tissue or in surrounding normal tissue,^36^ seropositivity to *H. pylori* infection was rarely found in the small subset of IPMN cases included in our study.

Contrary to our main research hypothesis, we observed inverse associations between certain *H. pylori* proteins and PC. Although these associations did not reach significance, they may be an indication of *H. pylori's* protective effect against PC. For example, the odds of PC among HP522-positive individuals were reduced by four times, the odds of PC among HyuA positives were reduced by two times, and the odds of PC among HP1564 positives were reduced by 1.6 times. Future studies should evaluate these inverse associations in a large sample size to generate more conclusive evidence. Several possible interpretations could describe these null findings, including *H. pylori* not playing a role in the etiology of PC. It is also possible that certain positive and negative associations observed in the current study did not reach statistical significance due to the small sample sizes that were evaluated. It would have also been preferable to have a more similar age distribution in the cases and controls. The International Agency for Research on Cancer (IARC) classified *H. pylori* as a cancer-causing agent in 1994.^37^ Since then, *H. pylori* have been recognized as an important cause of gastric cancer.^31,38^ However, several studies have reported an inverse relationship between *H. pylori* infection and cancer of the cardia region of stomach.^39–41^ Studies have also shown that regions with a high prevalence of *H. pylori* typically have high incidence rates of gastric cancer, and that the *H. pylori* eradication efforts in some areas of the world have led to reduction in gastric cancer rates.^31,42–44^ In the United States, those race/ethnic groups that suffer from high burden of *H. pylori* also suffer from a high burden of gastric cancer. For example, American Indians and Alaska Native (AI/AN) and African American populations have a high prevalence of *H. pylori* and higher rates of gastric cancer than the general U.S. population.^45–49^ Our study is limited in that we did not have the opportunity to validate our experimental design using samples from patients with gastric cancer. In contrast, PC cases per 100,000 individuals in the population are lower among AI/AN than whites (male: 13.4 vs. 15.0 and females: 8.2 vs. 11.7), and higher among African Americans than whites (male: 17.0 vs. 15.0 and females: 8.2 vs. 11.7).^50^ Small sample sizes in subgroups limited our ability to conduct stratified analyses by race, and type/location of the tumor and the role of *H. pylori* in the etiology of PC require further investigation. It is possible that *H. pylori* may modify the risk of PC in either direction, depending on the type and location of tumor, and its interaction with other potential risk factors,^51^ such as tobacco use, diabetes, obesity, dietary factors, alcohol use, and chronic pancreatitis and ABO blood group. Nevertheless, the etiology of PC is complex; the causes of pancreatic carcinoma are still not sufficiently known and require investigation.

Strengths of this study included evaluation of a comprehensive list of *H. pylori* proteins, a robust and standardized laboratory protocol, and inclusion of pathologically confirmed cases. However, the study may be limited because the use of a serologic test to determine infection status through assessment of circulating antibody levels does not enable discrimination between present and past infections. Antibodies against *H. pylori* proteins may stay in serum from several months to several years depending on whether the infection is treated or left untreated.^52,53^ While present infection to *H. pylori* may not play a role in pancreatic carcinogenesis, past infections may be important for oncogenesis. Also, inherent to all seroepidemiologic studies, the impact of antibody decay and seroconversion rate on the seroprevalence estimates cannot be evaluated. In this study *H. pylori* is not associated with PC. Further research is required to evaluate this association in large longitudinal studies with access to prediagnostic samples. The lack of association between *H. pylori* infection and PC risk has been inconclusive. Using a multiplex assay against 15 *H. pylori* antigens, we found no association between seropositivity to *H. pylori* and PC risk in a case-control study in the United States. Our results suggest that *H. pylori* may not be a risk factor for PC. Given the high prevalence of *H. pylori* in population, and the high mortality rate of PC, we recommend future longitudinal studies to further explore this association.

## Conclusion

The prevalence of H. *pylori* in the United States is 35.6%, with marked disparities (e.g., the prevalence in indigenous Alaskans population is close to 75%) making it an important public health concern. Similarly, PC remains one of the deadliest cancer types. Therefore, studying the role of *H. pylori* in the pathogenicity of PC has public health significance. We did not observe association between seropositivity to *H. pylori* and PC risk in a case-control study in the United States. Our study results do not support an association between *H. pylori* and PC. Considering the high prevalence of *H. pylori* in populations, and the high mortality rate of PC, we recommend future longitudinal studies to further explore this lack of association.

## Abbreviations Used

AI/AN: American Indians and Alaska Native
ATBC: Alpha-Tocopherol, Beta-Carotene Cancer Prevention Study
CagA: cytotoxin-associated gene A
CI: confidence interval
EDTA: ethylenediaminetetraacetic acid
ELISA: enzyme-linked immunosorbent assay
GST: glutathione-S-transferase
IARC: International Agency for Research on Cancer
IPMN: intraductal papillary mucinous neoplasm
IRB: Institutional Review Board
OR: odds ratio
PC: pancreatic cancer
TCC: Total Cancer Care
